# Supplementary material for: Non-invasive, opsin-free mid-infrared modulation activates cortical neurons and accelerates associative learning
Source: Nat Commun. 2021 May 12;12:2730. doi: 10.1038/s41467-021-23025-y (PMC8115038; doi:10.1038/s41467-021-23025-y)
Supplement: Supplementary file 1 — Supplementary Information [file 41467_2021_23025_MOESM1_ESM.pdf]

Supplementary Information

Non-invasive, opsin-free mid-infrared modulation activates cortical neurons and accelerates associative learning

Zhang et al.

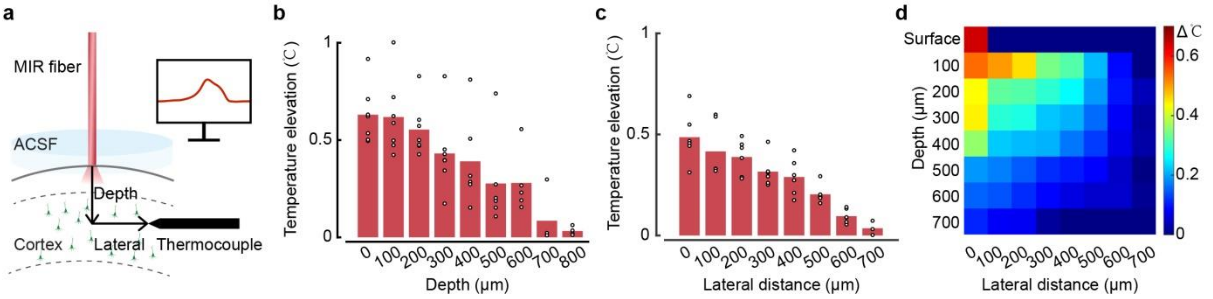

Supplementary Fig. 1 Temperature measurements in cortical tissue in vivo with MIM application. **a** Schematic illustrating the in vivo cortical tissue temperature measurements, the same parameters of MIM as those for in vivo physiological experiments (duration 20 s). **b** Temperature elevation (plateau of temperature elevation from baseline upon light irradiation) with respect to the depth of thermocouple probe (depth from tip of optical fiber on the central axis). *n* = 7 mice. **c** Similar to panel *b*, showing temperature elevation with respect to the lateral distance from the fiber tip (depth fixed at 100 μm). *n* = 6 mice. **d** Heatmap summarizing the spatial profile of temperature elevation upon MIM application. *n* = 6 mice.

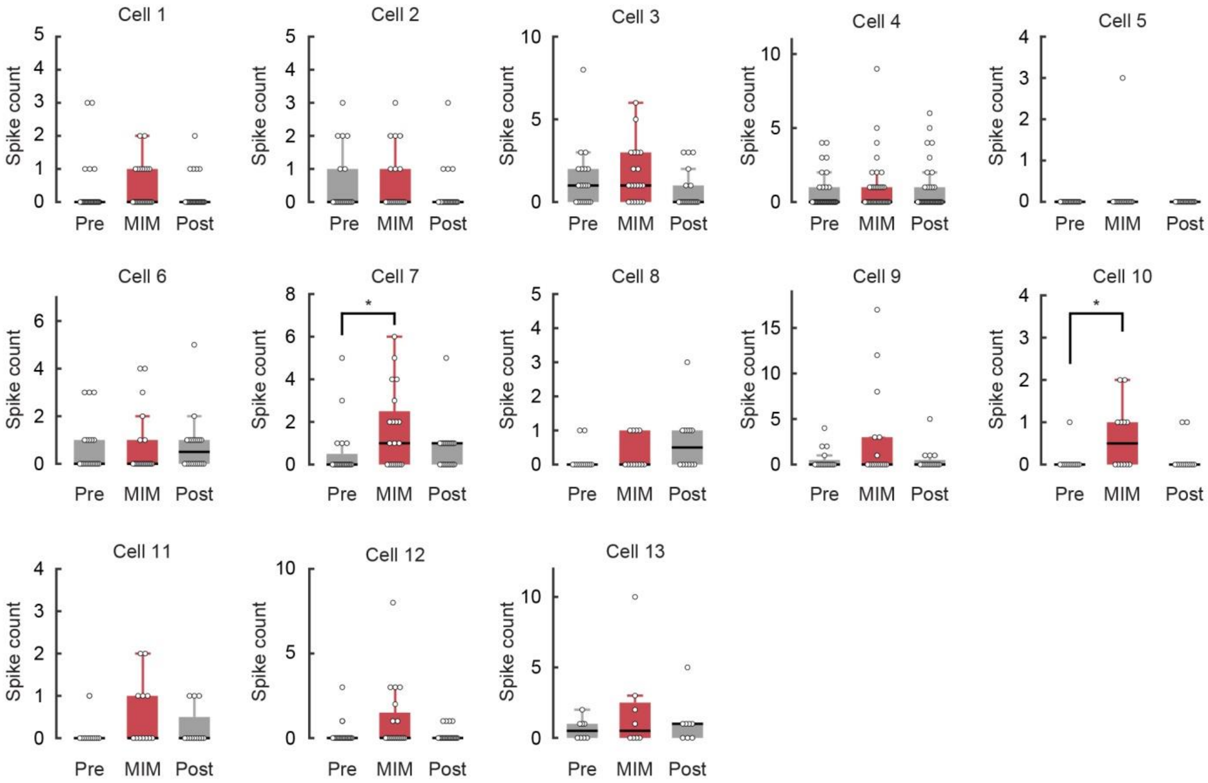

Supplementary Fig. 2 Loose-patch recording, cell-by-cell analysis. Each graph shows data analyzed for one of the 13 recorded cells. All recordings were split into time bins of 5 s each, and each data point is the spike count in a bin, categorized as ‘Pre’ (before MIM application window), ‘MIM’ (within the MIM application time window), ‘Post’ (after MIM application window). $P = 0.02$ (Cell 7, Pre vs MIM), $P = 0.027$ (Cell 10, Pre vs MIM), $^*P < 0.05$, two-sided Wilcoxon rank-sum test. The box-and-whisker plots indicate the median (central mark), 25th and 75th percentiles (bounds of box: Q1 and Q3), interquartile range (IQR: Q3-Q1), and the whiskers extending to the minima and maxima without considering outliers.

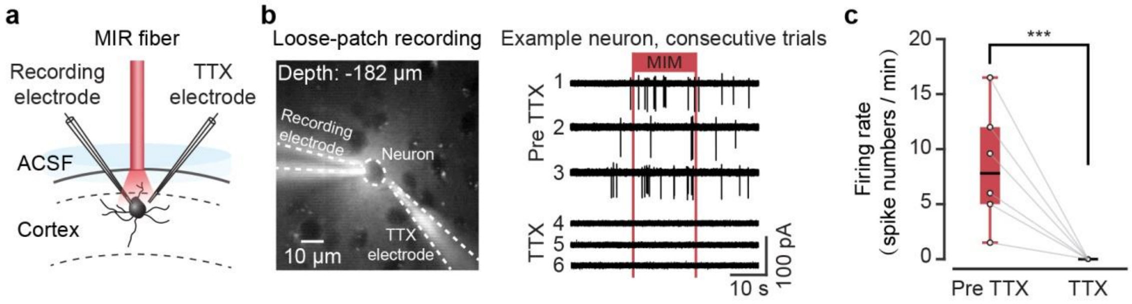

Supplementary Fig. 3 Loose-patch recording, TTX control experiment. **a** Schematic showing the in vivo loose-patch recording with TTX application. **b** Left, a two-photon image showing an example of how the loose-patch recording electrode and the TTX delivery electrode are positioned. Right, consecutive trials of recording from one example neuron showing spiking before and after TTX delivery (50 mbar, 10 s injection). **c** Boxplot summary of spike count before and after TTX delivery. $n = 6$ cells. $P = 0.00022$, two-sided Wilcoxon signed-rank test, ***$P < 0.001$. The box-and-whisker plots indicate the median (central mark), 25th and 75th percentiles (bounds of box: Q1 and Q3), interquartile range (IQR: Q3-Q1), and the whiskers extending to the minima and maxima without considering outliers.

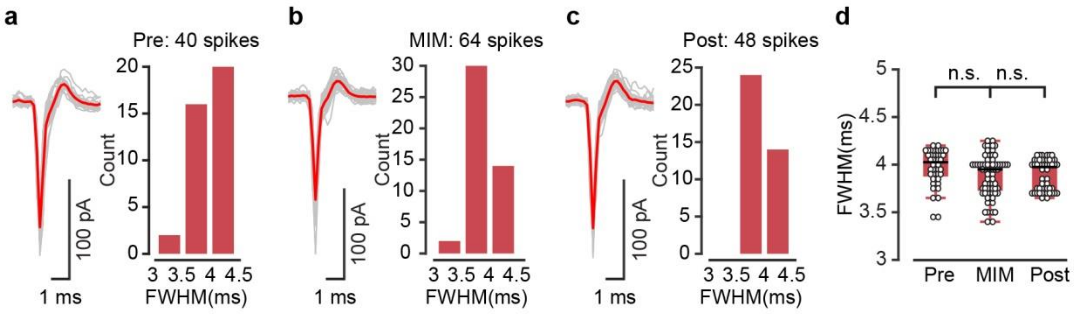

Supplementary Fig. 4 Loose-patch recording, spike waveform width analysis. a-c Left traces: overlay of sampled spike waveforms; right histogram: spike waveform width (full-width at half-maximum, FWHM); sampled before ('Pre'), during ('MIM') and after ('Post') MIM application, respectively. **d** Boxplot summary of spike width. n.s., $P > 0.05$, two-sided Wilcoxon rank-sum test. The box-and-whisker plots indicate the median (central mark), 25th and 75th percentiles (bounds of box: Q1 and Q3), interquartile range (IQR: Q3-Q1), and the whiskers extending to the minima and maxima without considering outliers.

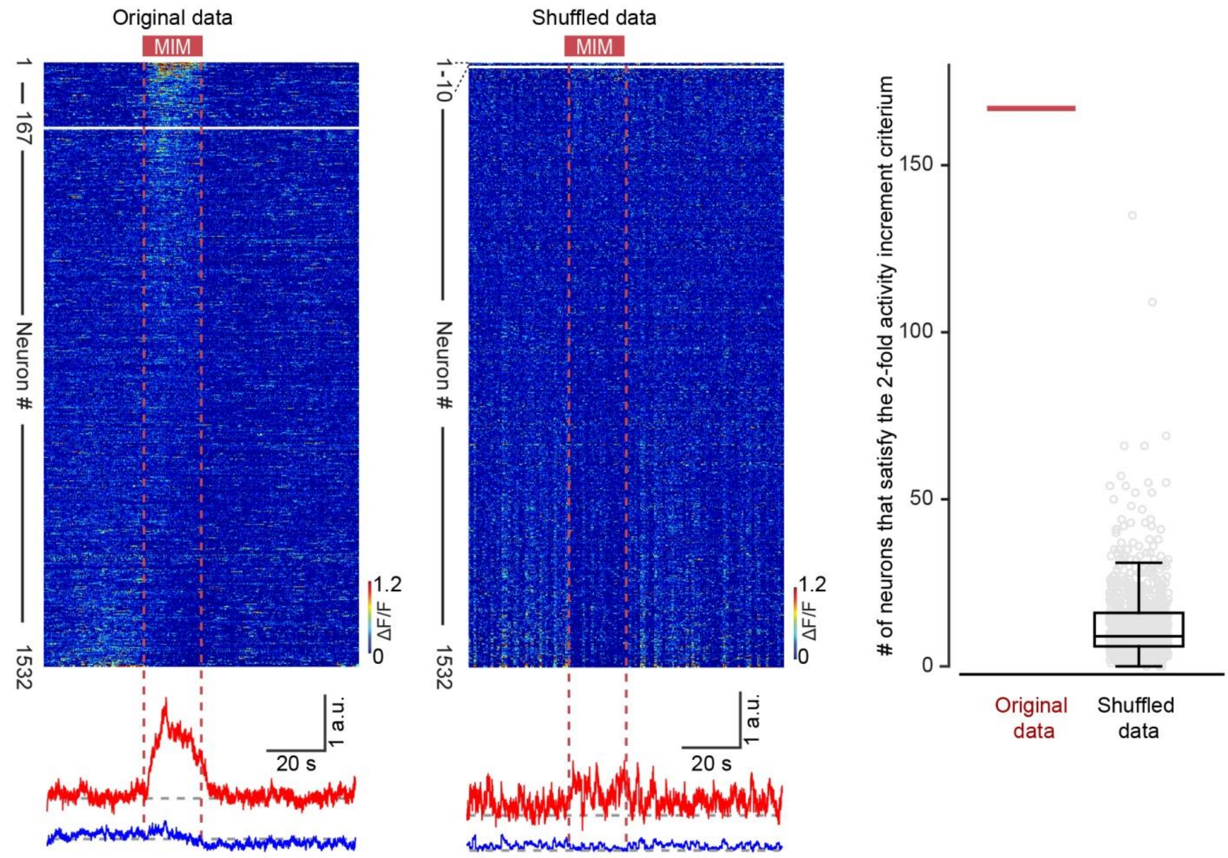

Supplementary Fig. 5 Complete dataset of the two-photon Ca^{2+} imaging experiments. Left: Pseudocolored map of trial-averaged Ca^{2+} activity of altogether 1532 cells which were pooled from recordings in 6 mice, and sorted by their relative increment of Ca^{2+} activity level from pre-MIM to MIM. The top 167 cells satisfied the '2-fold' criteria that defines a neuron as MIM-activated. Red trace below the map shows the grand average Ca^{2+} signal trace of the 167 MIM-activated neurons (same as in Fig. 2f), blue trace shows that of the rest 1365 neurons. **Middle**: The original data were divided into segments of 1 s, and then we randomly assigned the segments of Ca^{2+} data to shuffle the responses (1000 times). Here only 10 out of 1532 neurons were shown above the 2-fold threshold for the shuffled data. Red trace and blue trace are similar to those in Left panel. **Right**: The number of MIM-activated neurons for the original data (red) and the shuffled data (black). The number of the MIM-activated neurons was significantly greater than that of the randomly shuffled data (*P* = 3.99e-4, two-sided bootstrap test). The box-and-whisker plots indicate the median (central mark), 25th and 75th percentiles (bounds of box: Q1 and Q3), interquartile range (IQR: Q3-Q1), and the whiskers extending to the minima and maxima without considering outliers.
