# Supplementary material for: Non-invasive, opsin-free mid-infrared modulation activates cortical neurons and accelerates associative learning
Source: Nat Commun. 2021 May 12;12:2730. doi: 10.1038/s41467-021-23025-y (PMC8115038; doi:10.1038/s41467-021-23025-y)
Supplement: Supplementary file 3 — Reporting Summary [file 41467_2021_23025_MOESM3_ESM.pdf]

nature research

Corresponding author(s): Xiaowei Chen

Last updated by author(s): Mar 22, 2021

Reporting Summary

Nature Research wishes to improve the reproducibility of the work that we publish. This form provides structure for consistency and transparency in reporting. For further information on Nature Research policies, see our Editorial Policies and the Editorial Policy Checklist.

Statistics

For all statistical analyses, confirm that the following items are present in the figure legend, table legend, main text, or Methods section.

n/a	Confirmed	
☐	☒	The exact sample size (n) for each experimental group/condition, given as a discrete number and unit of measurement
☐	☒	A statement on whether measurements were taken from distinct samples or whether the same sample was measured repeatedly
☐	☒	The statistical test(s) used AND whether they are one- or two-sided<br>*Only common tests should be described solely by name; describe more complex techniques in the Methods section.*
☒	☐	A description of all covariates tested
☐	☒	A description of any assumptions or corrections, such as tests of normality and adjustment for multiple comparisons
☐	☒	A full description of the statistical parameters including central tendency (e.g. means) or other basic estimates (e.g. regression coefficient) AND variation (e.g. standard deviation) or associated estimates of uncertainty (e.g. confidence intervals)
☐	☒	For null hypothesis testing, the test statistic (e.g. F, t, r) with confidence intervals, effect sizes, degrees of freedom and P value noted<br>*Give P values as exact values whenever suitable.*
☒	☐	For Bayesian analysis, information on the choice of priors and Markov chain Monte Carlo settings
☒	☐	For hierarchical and complex designs, identification of the appropriate level for tests and full reporting of outcomes
☒	☐	Estimates of effect sizes (e.g. Cohen's d, Pearson's r), indicating how they were calculated

Our web collection on statistics for biologists contains articles on many of the points above.

Software and code

Policy information about availability of computer code

Data collection	We collected our data using LabVIEW 2012 (National Instruments) and PatchMaster v2x65 (HEKA Elektronik).
Data analysis	We analyzed our data using Igor Pro 5.0 (Wavemetrics) and Matlab 2014a (Mathworks).

For manuscripts utilizing custom algorithms or software that are central to the research but not yet described in published literature, software must be made available to editors and reviewers. We strongly encourage code deposition in a community repository (e.g. GitHub). See the Nature Research guidelines for submitting code & software for further information.

Data

Policy information about availability of data

All manuscripts must include a data availability statement. This statement should provide the following information, where applicable:
- Accession codes, unique identifiers, or web links for publicly available datasets
- A list of figures that have associated raw data
- A description of any restrictions on data availability

The data that support the findings of this study are available from the corresponding author upon reasonable request. Source data underlying Figs. 1–3 and Supplementary Figs. 1–5 are available as a Source data file. Source data are provided with this paper.

Field-specific reporting

Life sciences study design

All studies must disclose on these points even when the disclosure is negative.

Sample size	No statistical methods were used to predetermine sample sizes. Samples sizes were determined based on our own previous studies (Chen et al, Nature, 2011; Wang et al, Nature Communications, 2020) about using behavioral, two-photon imaging and electrophysiological recording approaches. Samples sizes adopted in this study were sufficient for detecting robust effects.
Data exclusions	No data was excluded from analysis.
Replication	All immunohistochemistry, behavioral, two-photon imaging and electrophysiological experiments were performed over at least 4 independent replicates. The study effects were all successfully replicated in the experiments.
Randomization	For all experiments, samples were randomized where appropriate for data collection and analysis.
Blinding	In the studies, investigators were blinded to groups for data collection and analysis.

Reporting for specific materials, systems and methods

We require information from authors about some types of materials, experimental systems and methods used in many studies. Here, indicate whether each material, system or method listed is relevant to your study. If you are not sure if a list item applies to your research, read the appropriate section before selecting a response.

Materials & experimental systems

n/a	Involved in the study	
☐	☒	Antibodies
☒	☐	Eukaryotic cell lines
☒	☐	Palaeontology and archaeology
☐	☒	Animals and other organisms
☒	☐	Human research participants
☒	☐	Clinical data
☒	☐	Dual use research of concern

Methods

n/a	Involved in the study	
☒	☐	ChIP-seq
☒	☐	Flow cytometry
☒	☐	MRI-based neuroimaging

Antibodies

Antibodies used	The immunostaining was performed with the following antibodies: Anti-c-Fos 1:500 (Millipore, ABE457, rabbit polyclonal antibody, Lot #3221531), Alexa Fluor 594 goat anti-rabbit IgG 1:500 (Invitrogen, A11012, Lot #2119134), NeuN 1:200 (Millipore, MAB377, mouse polyclonal antibody, Lot #3519281), Alexa Fluor 488 donkey anti-mouse IgG 1:500 (Invitrogen, A21202, Lot #1644644).
Validation	The application and specificity of all the antibodies are validated by the companies. The primary antibodies: Anti-c-Fos (ABE457, Millipore rabbit polyclonal antibody, Lot# 3221531, 1:500 dilution), Anti-NeuN (MAB377, Millipore, mouse polyclonal antibody, 1:200 dilution) were validated for both species and application by the manufacturer: (1) Anti-c-Fos, Cat. No. ABE457, is a rabbit polyclonal antibody that detects Proto-oncogene c-Fos and is tested for use in Immunohistochemistry (Paraffin), Peptide Inhibition Assay, and Western Blotting; (2) Anti-NeuN Antibody, clone A60 detects level of NeuN and has been published and validated for use in FC, IC, IF, IH, IH(P), IP and WB. Please visit the company’s website for details. Anti-c-Fos: https://www.merckmillipore.com/CN/zh/product/Anti-c-Fos-Antibody,MM_NF-ABE457; Anti-NeuN: https://www.merckmillipore.com/CN/zh/product/Anti-NeuN-Antibody-clone-A60,MM_NF-MAB377.

Animals and other organisms

Policy information about studies involving animals; ARRIVE guidelines recommended for reporting animal research

Laboratory animals	C57BL/6J male mice (2-3 months old) were provided by the Laboratory Animal Center of the Third Military Medical University. The mice were housed in a temperature- and humidity-controlled room on a cycle of 12 h light/dark (lights off at 19:00).
Wild animals	No wild animals were used in this study.
Field-collected samples	No field-collected samples were used in this study.
Ethics oversight	Third Military Medical University Animal Care and Use Committee.

Note that full information on the approval of the study protocol must also be provided in the manuscript.
